# Video-Based Fingerprint Verification

**DOI:** 10.3390/s130911660

**Published:** 2013-09-04

**Authors:** Wei Qin, Yilong Yin, Lili Liu

**Affiliations:** School of Computer Science and Technology, Shandong University, Jinan 250101, Shandong, China; E-Mails: weiqin_wq@163.com (W.Q.); ll_liu@aliyun.com (L.L.)

**Keywords:** fingerprint, fingerprint verification video, dynamic information, similarity, relative match score

## Abstract

Conventional fingerprint verification systems use only static information. In this paper, fingerprint videos, which contain dynamic information, are utilized for verification. Fingerprint videos are acquired by the same capture device that acquires conventional fingerprint images, and the user experience of providing a fingerprint video is the same as that of providing a single impression. After preprocessing and aligning processes, “inside similarity” and “outside similarity” are defined and calculated to take advantage of both dynamic and static information contained in fingerprint videos. Match scores between two matching fingerprint videos are then calculated by combining the two kinds of similarity. Experimental results show that the proposed video-based method leads to a relative reduction of 60 percent in the equal error rate (EER) in comparison to the conventional single impression-based method. We also analyze the time complexity of our method when different combinations of strategies are used. Our method still outperforms the conventional method, even if both methods have the same time complexity. Finally, experimental results demonstrate that the proposed video-based method can lead to better accuracy than the multiple impressions fusion method, and the proposed method has a much lower false acceptance rate (FAR) when the false rejection rate (FRR) is quite low.

## 1. Introduction

In ancient China and many other countries and districts, people had been aware that a fingerprint can be used for identity authentication [1]. However, it was not until 1880 that Henry Fauld first scientifically suggested the individuality of fingerprints based on empirical observations [2]. In 1892, Galton published the well-known book entitled *Fingerprints*, in which he discussed the basis of contemporary fingerprint science, including persistence, uniqueness and classification of fingerprints [3]. In the early twentieth century, fingerprint recognition was formally accepted as a valid personal identification method and became a standard routine in forensics [1]. With the rapid expansion of fingerprint recognition in forensics, operational fingerprint databases became so huge that manual fingerprint identification became infeasible, which led to the development of Automatic Fingerprint Identification Systems (AFIS) using a computer for fingerprint verification [2].

Fingerprint verification is one of the most popular and reliable biometric techniques for automatic personal identification [4]. Unlike the conventional possession-based (e.g., passport) or knowledge-based (e.g., passwords) identity authentication schemes, the fingerprint identifier cannot be misplaced, forgotten, guessed or be easily forged. During recent years, fingerprint verification has received more and more attention and has been widely used in both forensic and commercial applications. Despite the brilliant achievements it has made, its wider-scale deployment has been hindered, due to challenging problems in fingerprint verification technology.

The main challenge of fingerprint verification is its less than satisfactory accuracy in some application domains. To improve the accuracy of fingerprint systems, three aspects of work are undertaken. Firstly, researchers focus on improving the performance of one or more steps of AFIS based on a single impression. The steps include segmentation [5,6], enhancement [7–9], representation and feature extraction (minutia-based [10], Ridge-based [11,12], texture-based [13,14], correlation-based [15,16]), matching [17,18], *etc.* Secondly, researchers try to use multiple sources of fingerprints to access higher accuracy. These sources include multiple biometric traits [19,20], multiple sensors [21], multiple representations and matchers [13,22,23], multiple fingers [23] and multiple impressions of the same finger [24–26]. Thirdly, new features are explored for matching beyond traditional features. Level 3 features, such as pores and ridge contours extracted from high resolution fingerprint images, are employed for fingerprint recognition, and the performance gain by introducing level 3 features is also studied [3,27–30].

All of these methods use *static information* (information from one static impression or from several temporal-independent static impressions), and no *dynamic information* (information from a video) is introduced. Dorai *et al.* [31,32] acquired a fingerprint video while a finger was interacting with the sensor. Then, they detected the distortion of fingerprint impressions due to excessive force and the positioning of fingers during image capture. They also investigate two aspects of dynamic behaviors from video and propose a new type of biometrics, named “resultant biometrics”. This offers us the enlightenment that we can use videos for fingerprint verification to achieve higher accuracy. Dorai *et al.* [31,32] focused on detecting distortion generated during the impression acquisition process and investigated the transformation of a user's biometrics over time. However, they did not directly use video for fingerprint verification.

**Figure 1.**
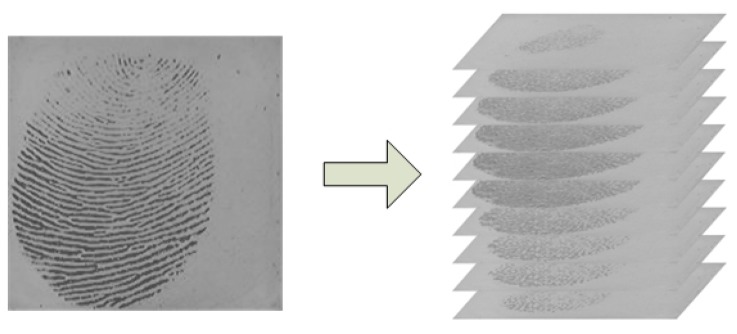
From static image (a single impression) to video (a fingerprint video).

In recent years, hardware technologies have matured to the point that we are able to transmit, store, process and view video signals that are stored in digital formats [33]. In fact, most of the currently used fingerprint capture devices have the capability to acquire fingerprint videos if the capturing software is modified accordingly. In the book [33], Bovik states that “*this* (*from static image to video*, see Figure 1) *is a natural evolution, since temporal change, which is usually associated with motion of some type*, *is often the most important property of a visual signal*”. Firstly, from fingerprint video, we can explore more useful information, which can be used to improve the accuracy of fingerprint verification. Secondly, the user experience of providing a fingerprint video is the same as that of providing a single impression. Thirdly, there are many ways a hacker can obtain fingerprint data of a specific user. For example, a latent fingerprint left on physical surfaces that the user has touched can be lifted and used for attacking a fingerprint system. However, the fingers' pressing process and the dynamic behaviors contained in the process cannot be left on the surface. Therefore, there may be potential benefits of using fingerprint video with respect to alleviating security issues. Therefore, investigating video-based fingerprint verification is meaningful and interesting work.

This paper is a significant extension of an earlier and much shorter version presented in [34]. The rest of this paper is organized as follows: In Section 2, we give analysis of a fingerprint video. Section 3 presents one video-based fingerprint verification method. Section 4 describes the experimental procedure and presents the experimental results. Finally, conclusions and future work are given in Section 5.

## 2. Fingerprint Video Analysis

### 2.1. Fingerprint Video Capturing

Nowadays, video capturing technology is mature enough to be able to deliver images at a relative high frame rate and the frame rate can be adjusted according to the demands of applications. During a capturing procedure, we can acquire a fingerprint video that records the whole process, from a finger touching the sensor surface to the finger leaving the surface. Actually, many single impression capture devices that use the touch method (non-sweep method) have the capability to generate images at a certain time interval. However, only one impression that satisfies some conditions is saved. In the same scenario, a fingerprint video can be acquired if we save more impressions in the capturing procedure. Therefore, there is no difference between providing a single impression and providing a fingerprint video from the user's point of view. The capture device and time cost of acquiring a single impression and a fingerprint video are the same.

### 2.2. Dynamic Information

A fingerprint video consists of a sequence of fingerprint impressions. On the one hand, impressions in a fingerprint video vary, due to distortion, deformation and the changing of the fingerprint area. On the other hand, there is strong correlation between fingerprint impressions inside a video, if there is no significant distortion and deformation. It can be inferred that the match score between two impressions in the same fingerprint video should be quite high.

One advantage of using video is that we can select the impression with better image quality, e.g., the impression with the largest fingerprint area. The other advantage is that there exist impressions different from each other in a video. Therefore, more information is introduced, and fusion methods can be taken to use these impressions for higher accuracy. These all make use of *static information*. More importantly, we can also take advantage of the strong correlation between impressions inside a fingerprint video, which is a kind of *dynamic information*.

### 2.3. Fingerprint Video Versus Multiple Impressions

**Figure 2.**
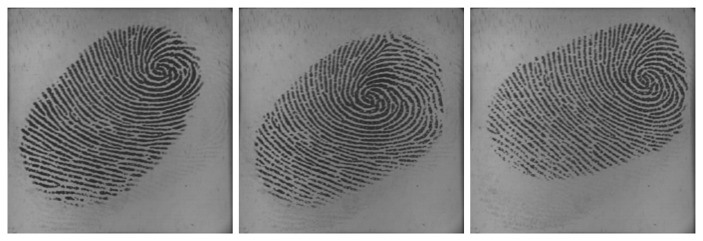
Three impressions from the same finger.

**Figure 3.**
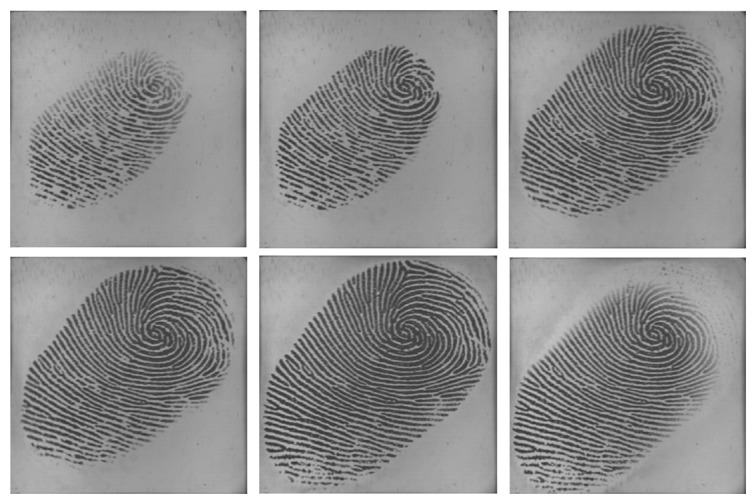
Six impressions in a fingerprint video. The fingerprint area of the first one enlarges gradually and, then, decreases.

Utilizing multiple impressions from the same finger has been proven to be effective to improve the accuracy of fingerprint systems [25,26]. Fingerprint video also contains a sequence of impressions; however, multiple impressions and fingerprint video are quite different. Firstly, multiple impressions of the same finger are acquired in multiple independent capturing procedures, while a fingerprint video is acquired in one capturing procedure. Secondly, multiple impressions of the same finger are relatively independent, *i.e.*, they may capture different regions of the finger. Even if they capture the same region of the finger, the signal-to-noise ratio may be quite different. However, impressions in a fingerprint video have strong correlation, as they are temporal-dependent and vary gradually. The strong correlation is a kind of dynamic information, which can be used to improve fingerprint verification accuracy. Figures 2 and 3 give examples of multiple impressions of the same finger and a fingerprint video, respectively.

## 3. Video-Based Fingerprint Verification Method

**Figure 4.**
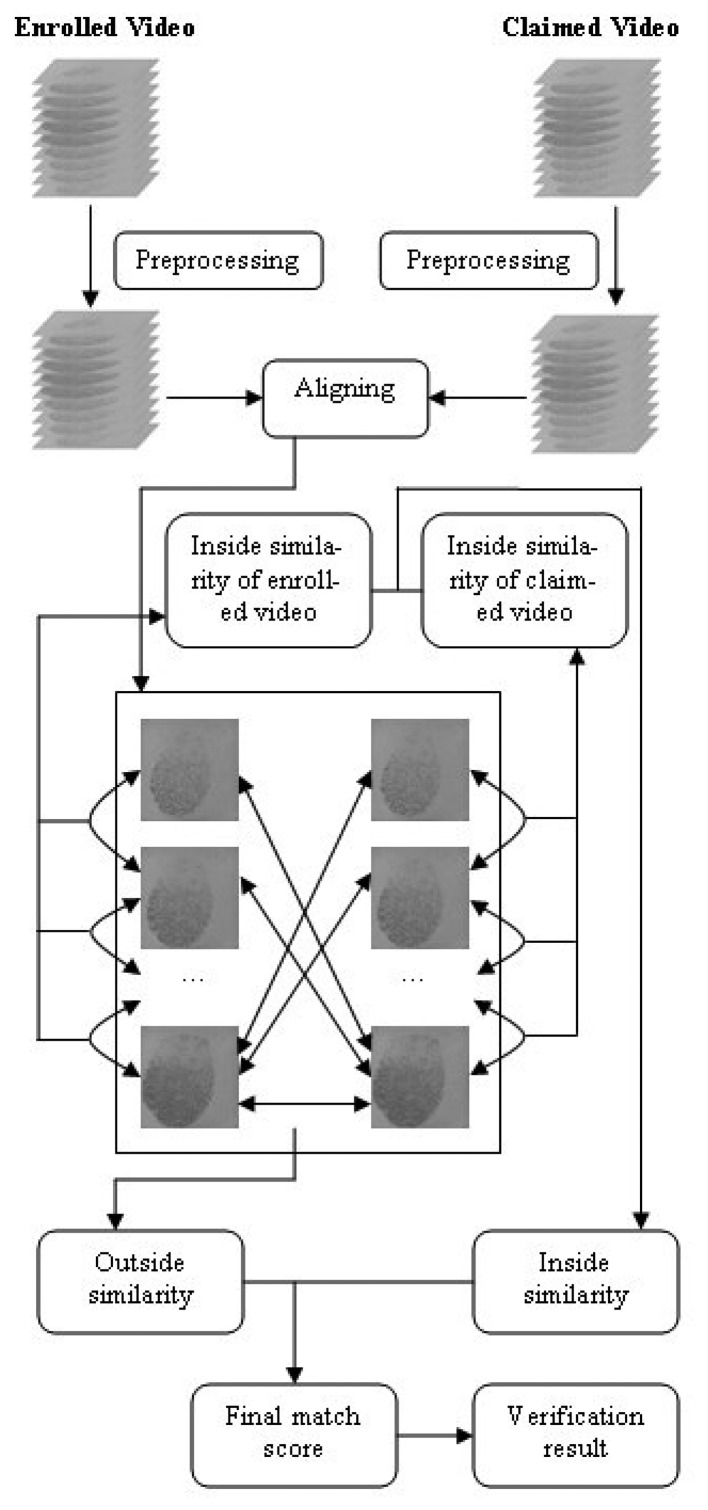
Flow chart of the video-based fingerprint verification method.

The proposed video-based fingerprint verification method contains the following steps: video preprocessing, videos aligning, calculating the inside similarity, calculating the outside similarity, combining the two kinds of similarities and, finally, verification. The flow chart of the schemes is shown in Figure 4.

### 3.1. Preprocessing of Fingerprint Video

The raw enrolled fingerprint videos cannot be used for verification directly, as there may exist fingerprint images that have limited benefits (such as impressions with too small of a fingerprint area) for recognition. Additionally, the computational cost will be reduced if fewer images are used for verification. Therefore, a preprocessing step is needed to select fingerprint images that will be used for verification. There are many rules to select effective fingerprint images, *i.e.*, foreground size and gray-value contrast [35]. For different purposes, researchers choose different selection criteria. For example, for a system using a single impression for verification, the image should be large and clear; for a system using multiple impressions, the diversity of the impressions should be considered. Considering that a fingerprint video contains many adjacent duplicate images, reserving one copy of them will not lose any useful information. Besides, as a fingerprint video has strong correlation, which is beneficial for verification, we have to reserve the continuity of the chosen images. Therefore, in this paper, the foreground size is applied as the criterion to decide which image should be reserved in a fingerprint video. It should be noted that the adjacent images, which have the same foreground size, are considered as duplicate images, and the foreground size is measured by foreground blocks. The process of determining the reserved images is illustrated as follows:

Suppose the set of fingerprint images in an enrolled fingerprint video is represented as:

(1) $$R=\{F_{i}^{R}|i=1,2,\ldots m\}$$

where *m* is the number of images in the fingerprint video and $F_{i}^{R}$ is the ith image. First, each fingerprint image, $F_{i}^{R}$, in the raw video is segmented under the block-level using the segmentation method in [36], and the number of blocks in the foreground of $F_{i}^{R}$ is represented by *FP_Block_Num_i_*. Images with a *FP_Block_Num_i_* value smaller than a certain threshold, λ, will be abandoned. Besides, adjacent images with the same number of foreground blocks are considered as the same image, and only one of them will remain. After preprocessing, the set of remaining fingerprint images in the enrolled fingerprint video is represented as:

(2) $$E=\{F_{i}^{E}|i=1,2,\ldots n\}$$

where *n*(*n* ≤ *m*) is the number of remaining images and $F_{i}^{E}$ is the ith image. The image with the largest fingerprint area in this sequence is represented as $F_{\text{max}\_e}^{E}(1\leq \text{max}\_e\leq n)$. The remaining images are then used for verification. The preprocessing algorithm is summarized in Algorithm 1.

Similarly, we can also get the set of fingerprint images in the claimed video after preprocessing:

(3) $$C=\{F_{i}^{C}|i=1,2\ldots l\}$$

where *l* is the number of images and $F_{i}^{C}$ is the *i*th image. The image with the largest fingerprint area in this sequence is represented as $F_{\text{max}\_c}^{C}(1\leq \text{max}\_c\leq l)$.



**Algorithm 1** Fingerprint video preprocessing algorithm.
Input: Image set *R* and *E, FP_Block_Num_i_*(*i*=1, 2,…, *m*), threshold λProcedure:1.Set: *E* = Ø, *FP_Block _Num*_0_= -1, *j* = 12.for (*i*=1 to *m*)3. if (*FP_Block_Num_i_* ≥ λ and *FP_Block_Num_i_*≠*FP_Block_Num*_*i*−1_)4.  
$F_{i}^{R}$ is inserted into set *E* and represented as
$F_{j}^{E}$;5.  *j*=*j* + l;Output: Set *E*


### 3.2. Aligning Algorithm

Considering the computational complexity, we have to use the fewest impressions in a fingerprint video to get as high an accuracy as possible. Here, we propose an aligning method to reduce the number of impressions according to the characteristics of fingerprint videos.

**Figure 5.**
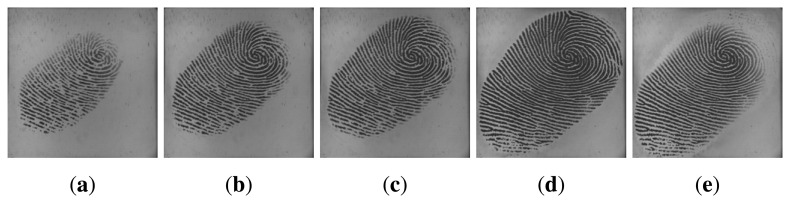

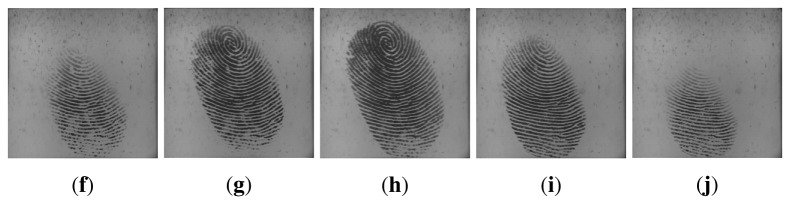
An example of our aligning method. (**a**-**e**) are impressions in the enrolled video after preprocessing; (**f**-**j**) are impressions in the claimed video after preprocessing. Impression (**d**) and impression (**h**) are images with the largest fingerprint area in the enrolled and claimed videos, respectively. Impressions (**b**-**e**) correspond to (**f**-**i**), while impressions (**a**,**j**) have no correspondences. After aligning, impressions (**a**,**j**) will be abandoned.

Generally speaking, from the finger contacting the sensor surface to the finger leaving the surface, the fingerprint area of the impressions first enlarges gradually and then decreases gradually, as shown in Figure 3. The impression with the largest fingerprint area could be seen as the “datum point”. Suppose there is a pair of matching videos: the frame sequences after preprocessing are *E* and *C*, as described in Equations (2) and (3), respectively. We select $F_{\text{max}\_e}^{E}$ and $F_{\text{max}\_c}^{C}$ as datum impressions and let $F_{\text{max}\_e}^{E}$ correspond to $F_{\text{max}\_c}^{C}$, $F_{\text{max}\_e-j}^{E}$ correspond to $F_{\text{max}\_c-j}^{C}$ (*j* = 1, 2, ⋯, *min*{*max_e*, *max_c*} − 1; and $F_{\text{max}\_e+j}^{E}$ correspond to $F_{\text{max}\_c+j}^{C}$ (*j* = 1, 2, ⋯, *min*{*n* − *max_e*, *m* − *max_c*}). Impressions that have no correspondences will not be used for verification. After aligning, there will be the same number of remaining impressions in both videos. An example of our aligning method is shown in Figure 5.

### 3.3. Inside Similarity and Outside Similarity

To use fingerprint videos for verification, we must define the similarity between two matching videos. The match score is used to measure the similarity between two videos. The proposed method uses both “inside similarity” and “outside similarity” to calculate the final match score between two videos. Outside similarity is calculated in the same way as the fusion method using multiple impressions of the same finger, and thus, the static information of a fingerprint video is utilized. However, the innovation of this paper is reflected in the definition of inside similarity, which takes advantage of the dynamic information of a fingerprint video. The proposed video-based method has been named Video Matching Score Calculation (VMSC), as it defines and calculates the match score between fingerprint videos.

There are two stages in fingerprint verification: enrollment and verification. During the enrollment stage, fingerprint videos are captured and stored as templates. Then, the inside similarity of each enrolled video can be calculated. During the verification stage, a new fingerprint video is acquired and compared to a stored template to verify whether they are from the same finger. In this stage, outside similarity is calculated, and the inside similarity of the claimed video can also be calculated.

#### 3.3.1. Inside Similarity

After aligning, the sequence of remaining impressions in an enrolled fingerprint video *V* can be represented as:

(4) $$E^{\prime }=\{F_{i}^{E}|i=1,2,\ldots k\}$$

where *k* is the number of impressions and $F_{i}^{E}$ is the *i*th impression; the sequence of remaining impressions in the claimed fingerprint video, *V'*, which matches against *V*, can be represented as:

(5) $$C^{\prime }=\{F_{i}^{C}|i=1,2,\ldots k\}$$

where *k* is the number of impressions and $F_{i}^{C}$ is the ith impression.

In the enrollment stage, we can calculate the match score, *S^E^*, which represents the inside similarity of the enrolled video. In the verification stage, match score *S^C^*, which represents the inside similarity of the claimed video, can be calculated. Here, we select two strategies to calculate *S^C^* according to different time complexity:

(1) **Strategy IS-1:**
  (6) $$S^{C}=\frac{1}{k-1}\sum_{i=1}^{k-1}S_{i,i+1}^{C}$$
  where $S_{i,i+1}^{C}$ is the match score between $F_{i}^{C}$ and $F_{i+1}^{C}$.
(2) **Strategy IS-2:**
  (7) $$S^{C}=\frac{1}{k*(k-1)/2}\sum_{j=1}^{k-1}\sum_{i=j+1}^{k}S_{i,j}^{C}$$
  where $S_{i,j}^{C}$ is the match score between $F_{i}^{C}$ and $F_{j}^{C}$.

*S^E^* can also be calculated by the two strategies described in Equations (6) and (7). It is worth noting that the calculation of *S^E^* is offline, and thus, the time complexity of calculating *S^E^* is not a main issue. The final inside similarity represented by match score *S^I^* can be calculated as:

(8) $$S^{I}=S^{E}$$

or:

(9) $$S^{I}=(S^{E}+S^{C})/2$$

according to different time complexity.

We also have to notice that the fingerprint area of impressions may be quite different; so, correspondingly, the number of minutia in a pair of matching impressions may vary greatly. In order to eliminate the effect of this difference, the following equation is used to calculate the match score between a pair of impressions:

(10) $$\text{score}=\frac{\text{num}\_\text{succ}}{\text{min}\{\text{num}\_1,\text{num}\_2\}}$$

where *num_succ* is the number of matched minutia and *num_*1 and *num_* 2 are the number of minutia in the two impressions, respectively. This equation will be used in all of the one-on-one matches in this paper.

#### 3.3.2. Outside Similarity

In the verification stage, outside similarity represented by the match score, *S^O^*, can be calculated. We select two strategies to calculate *S^O^* according to different time complexity:

(1) **Strategy OS-1:**
  (11) $$S^{O}=S_{\text{max}\_e,\text{max}\_c}$$
  where *S_max_e_*,*_max_c_* is the match score between $F_{\text{max}\_e}^{E}$ and $F_{\text{max}\_c}^{C}$.
(2) **Strategy OS-2:**
  (12) $$S^{O}=\frac{1}{2k}(\sum_{i=1}^{k}S_{i,\text{mac}\_c}+\sum_{j=1}^{k}S_{\text{max}\_e,j})$$
  where *S_i,max_c_* is the match score between $F_{i}^{E}$ and $F_{\text{max}\_c}^{C}$ (1 ≤ *i* ≤ *k*) and *S_max_e,j_* is the match score between $F_{\text{max}\_e}^{E}$ and $F_{j}^{E}$ (1 ≤ *j* ≤ *k*).

### 3.4. Combination of Inside and Outside Similarity

Considering an enrolled fingerprint video, *V*, and a claimed fingerprint video, *V*', the inside similarity and outside similarity are represented by *S^I^* and *S^O^*, respectively If this is a genuine match, outside similarity can be represented by $S_{g}^{O}$; if this is an impostor match, outside similarity can be represented by $S_{i}^{O}$.

There is *a priori* information that all the matches between two impressions in the same fingerprint video are genuine. As described in Section 2.2, the match score between two impressions in the same fingerprint video can be quite high, due to their strong correlation. Therefore, for a genuine match, inside similarity, *S^I^*, is an approximate representation of the maximum value of the outside similarity, $S_{g}^{O}$. Thus, if *S^I^* is not high, we have no reason to expect $S_{g}^{O}$ to be much higher than *S^I^*. Then, a *relative match score*, ∆*S*, can be introduced to improve the accuracy of verification.

Suppose ∆S = S^O^ - S^I^:

(1) if ∆*S* ≥ 0, the larger ∆*S* is, the more certain *V'* is genuine;
(2) if ∆*S <* 0, the larger the absolute value of ∆*S* is, the more certain *V'* is an impostor. For a genuine match, although the *absolute match score*, $S_{g}^{O}$, may be too low to lead to false rejection, the *relative match score*, ∆*S*, may be high enough to lead to correct verification. Table 1 shows the benefits of using the *relative match score* for verification.

In summary, the larger ∆*S* is, the more *V'* is certain to be genuine. Therefore, ∆*S* can be used to measure the similarity between two matching videos.

We propose to calculate the final match score, *S*, between two matching videos as follows:

(13) $$S=S^{O}+f(\Delta S)=S^{O}+f(S^{O}-S^{I})$$

where *f* (●) is an increasing function. We can use the simplest form as follows:

(14) $$S=S^{O}+\omega *\Delta S=S^{O}+\omega *(S^{O}-S^{I})$$

where *ω* is the weight of ∆*S* and *ω* > 0.

We have to notice that the foundation of this method is that the match score between two impressions in a same fingerprint video is quite high due to their strong correlation and *S^I^* is an approximate representation of the maximum value of $S_{g}^{O}$.

## 4. Experiments and Analysis

### 4.1. Database

**Figure 6.**
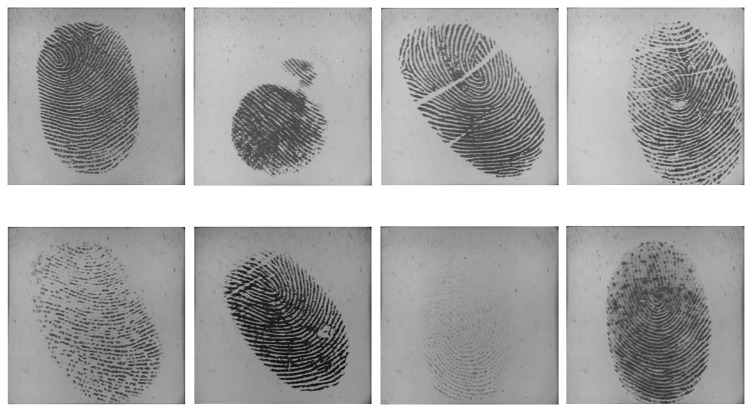
Sample fingerprint images in the SDU-FV database with various types and quality.

We collected fingerprint videos from 50 individuals using an optical fingerprint capture device. The frame rate is 25 frames/sec, and the frame size is 400 × 400 pixels with 72 dpiand 256 gray levels. The subjects mainly consisted of volunteers from the students and staff at Shandong University. Therefore, the database was named SDU-FV database. There were 20 females and 30 males in this database. Each volunteer provided 10 fingerprint videos from the same finger, and our database contained a total of 500 (50 × 10) videos. During the data acquisition process, we did not supervise or assist the subjects, in order to simulate the real situation as best as possible. Additionally, the subjects were not informed that they were providing fingerprint videos. The fingerprint images in the SDU-FV database vary in quality and type, including incompleteness, creases, scars and smudges in the ridges or dryness and blurs of the fingers. Some fingerprint samples are listed in Figure 6.

To our knowledge, the NIST24 database is the only public database of fingerprint videos. However, the fingerprint videos in this database are with deliberate distortions and deformations, which may lead the foundation of our method to not be satisfied. Moreover, the purpose of distributing the NIST 24 database is to determine how well the system tolerates significant plastic distortions, not to directly use videos for verification, which is quite different from the purpose of this paper. Therefore, the NIST 24 database is not suitable for testing our video-based method.

### 4.2. Analysis of the Proposed Method

#### 4.2.1. Data and Its Distribution

**Figure 7.**
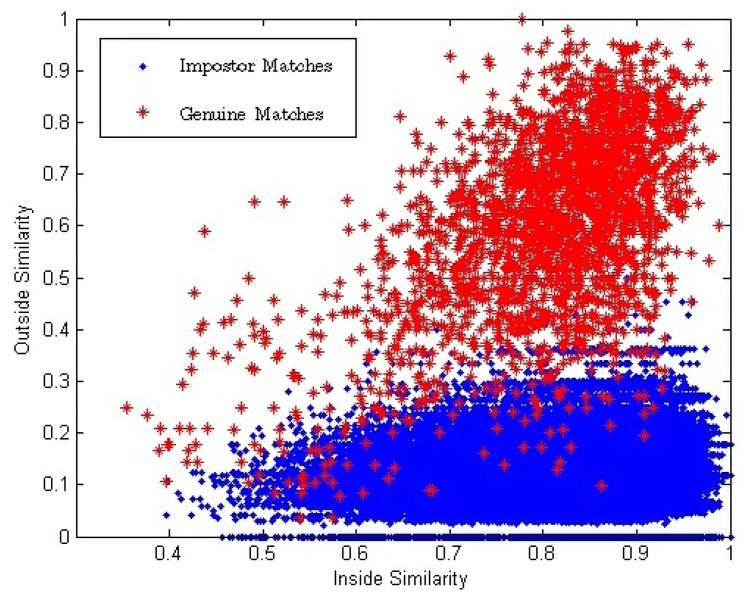
Two-dimensional distributions of (*S^I^*, *S^O^*) for all the genuine and impostor matches.

In the conventional single impression-based verification method, the similarity between two matching impressions used to make the final match decision is one-dimensional. In this paper, inside similarity, *S^I^*, and outside similarity, *S^O^*, are calculated and, thus, a two-dimensional similarity (*S^I^*, *S^O^*) between a pair of matching videos is introduced. Figure 7 shows the two-dimensional distributions of (*S^I^*, *S^O^*) for all the genuine and impostor matches.

Figure 7 shows that with the decrease of *S^I^*, both $S_{i}^{O}$ and $S_{g}^{O}$ also decrease. We use *E*(●) to represent the mathematical expectation. Because there exists strong correlation between the impressions inside a fingerprint video, we can conclude that:

(15) $$E(S_{i}^{O})<E(S_{g}^{O})<E(S^{I})$$

Therefore, $S_{i}^{O}$ and $S_{g}^{O}$. decrease with the decreasing of *S^I^*.

#### 4.2.2. Analysis of Proposed Equation

**Figure 8.**
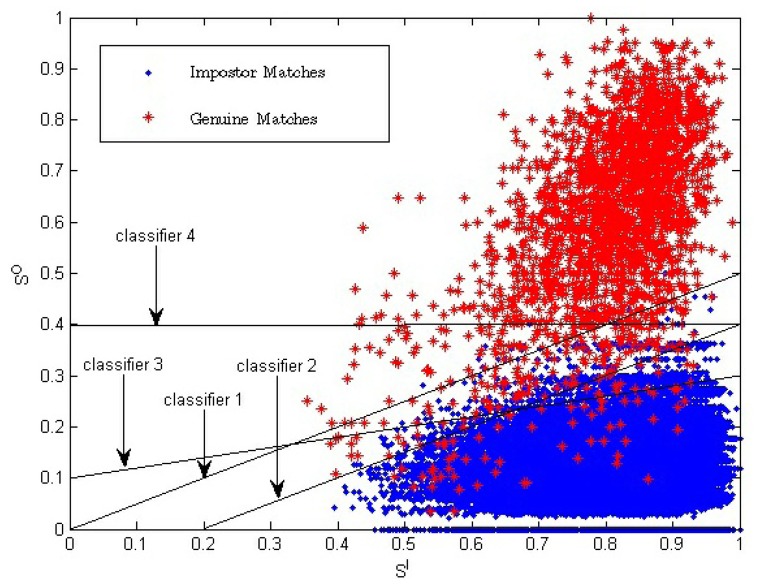
Example of classifiers of the proposed video-based method and the single impression-based method. Classifiers 1 to 3 are examples of the classifiers used in the proposed method. The ω values are 1.0, 1.0 and 4.0, respectively, and the threshold values of *Z* are zero, −0.2 and 0.5, respectively. Classifier 4 is an example of a classifier used in the single impression-based method.

Equation (14), which is proposed to combine inside and outside similarity, is equal to *Z* = *S^O^* + *ω* * (*S^O^* − *S^I^*) = (1 + *ω*) * *S^O^* − *S^I^*. In fact, in two-dimension space (*S^I^*, *S^O^)*, Equation (14) is a linear classifier which is determined by two parameters: the slope and the value of *Z*. The slope of the linear classifier is 1/(1 *+ ω*), and the value of *Z* is the chosen threshold that is used to get the final verification result. Examples of a linear classifier in two-dimension space (*S^I^*, *S^O^*) are given in Figure 8. Classifiers 1 to 3 are three examples with a different *ω* value or a different *Z* value.

For the single impression-based method using only one-dimensional similarity, *S^O^*, the classifier used to determine the verification result is the one-dimensional chosen threshold. In two-dimension space, the classifier can be represented as a line with the slope value of zero. Classifier 4 is an example, which is also shown in Figure 8.

#### 4.2.3. When and Why is the Proposed Method Effective

*S^I^* has positive correlation with fingerprint image quality. Suppose the value range of the match score is between zero and one. Let *P_g_* be the probability of a genuine match score being one, which represents the high genuine match score probability. Let *P_i_* be the probability of the impostor match score being one, which represents the high impostor match score probability. When *S^I^* is high, fingerprint image quality is, respectively, high. Therefore, the fingerprint image will have, respectively, clearer ridges and more fingerprint minutia. As a result, *P_g_* and *P_i_* will both be higher compared to the probability with lower fingerprint image quality. With the decreasing of *S^I^*, fingerprint image quality becomes lower, and *P_g_* and *P_i_* will become lower, too. The above analysis is consistent with the data distribution shown in Figure 8. The video-based method introduces another dimension compared to the traditional method. The traditional fingerprint verification method is equal to using Classifier 4, while the proposed method in this manuscript is equal to using Classifier 1 to Classifier 3; so, better verification results are acquired.

From Figure 8, we can see that classifiers corresponding to our proposed method have a better effect, especially in the region of *S^I^* < 0.7.

### 4.3. Verification

To measure the verification accuracy of our video-based method, each of the fingerprint videos is matched with all the other videos in the database. For the 50 × 10 videos, there will be a total number of 124, 750 matches, with 2, 250 genuine matches and 122, 500 impostor matches. After preprocessing, the number of frames in a video is 8.8 on average, and after aligning, the average number of frames decreases to six.

The minutiae-based matching method proposed in [10] is used for completing one-on-one matching. The minutiae-based method is a classic fingerprint verification method, which has profound influence in the fingerprint research area. The performance of a fingerprint verification system is mainly described by two values, *i.e.*, false acceptance rate (FAR) and false rejection rate (FRR). FAR and FRR are defined as:

(16) $$\text{FAR}=P(D_{1}|\omega_{2})$$

and:

(17) $$\text{FRR}=P(D_{2}|\omega_{1})$$

where *ω*_1_ and *ω*_2_ represent the classes of true genuine matches and impostor matches, respectively, and *D_1_* and *D_2_* denote the decisions of genuine matches and impostor matches, respectively. The equal error rate (EER) is computed as the point where FAR = FRR.

#### 4.3.1. Effect of Outside Similarity and Inside Similarity

In this section, we would like to study the effect on verification accuracy by using only outside similarity and the effect by introducing inside similarity.

**Figure 9.**
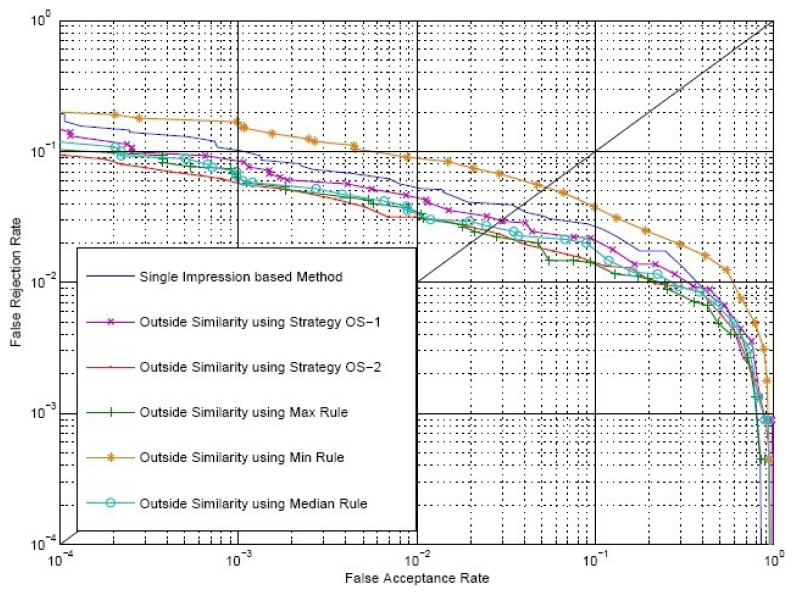
Receiver operating curves (ROC) of *S^R^* and *S^O^*.

Firstly, we carry out verification only using outside similarity, *S^O^*, which is calculated by Strategy OS-1 and Strategy OS-2, respectively. In order to make a more comprehensive contract, we also calculate outside similarity with the maximum rule, the minimum rule and the median rule, which are defined in Equations (18-20). The match scores of these three rules are represented by *S_max_*, *S_min_* and *S_med_*, respectively. As a reference for comparison, experiments using the single impression-based method are also carried out. We choose $F_{i}^{E}$ and $F_{j}^{C}$ from two matching impression sequences to calculate the single impression-based match score, *S^R^*, where *i* = *j* = (1 + *k)*/2, and *k* is the number of impressions in each of the aligned matching videos. Figure 9 shows the receiver operating curves (ROC) of *S^R^* and *S^O^* on the SDU-FV database plotting FAR *versus* FRR. The EER of using *S^R^* and *S^O^* calculated by Strategy OS-1, Strategy OS-2, maximum rule, minimum rule and median rule are 3.65%, 2.95%, 2.33%, 2.28%, 5.14% and 2.58%, respectively.

(18) $$S_{\text{max}}=\text{max}(S_{1,\text{max}\_c},\cdots ,S_{k,\text{max}\_c},S_{\text{max}\_e,1},\cdots ,S_{\text{max}\_e,k})$$

(19) $$S_{\text{min}}=\text{min}(S_{1,\text{max}\_c},\cdots ,S_{k,\text{max}\_c},S_{\text{max}\_e,1},\cdots ,S_{\text{max}\_e,k})$$

(20) $$S_{\text{max}}=\text{med}(S_{1,\text{max}\_c},\cdots ,S_{k,\text{max}\_c},S_{\text{max}\_e,1},\cdots ,S_{\text{max}\_e,k})$$

Secondly, the *k*-nearest neighbor (*k*-NN) method is used to test whether the two-dimensional similarity (*S^I^*, *S^O^*) can lead to better performance than the one-dimensional similarity, *S^O^*. We treat each pair of matching fingerprint videos as an instance; the inside similarity and outside similarity are considered as two features (*i.e.*, the *S^I^* feature and *S^O^* feature) of an instance. The instance labels are assigned as one, for genuine matching pairs, and zero, for impostor matching pairs. We then test the *k*-NN error rates using the S^O^ feature only and using two-dimensional features, *i.e.*, (*S^I^*, *S^O^*), respectively. Ten-fold cross-validation is employed to obtain unbiased error estimation. Three strategies are used to calculate *S^I^*: (i) using only *S^E^* to calculate *S^I^* by Equation (8); (ii) using Strategy IS-1 to calculate *S^C^*, and *S^I^* is calculated by Equation (9); and (iii) using Strategy IS-2 to calculate *S^C^*, and *S^I^* is calculated by Equation (9). S^E^ is calculated by Strategy IS-2 in all these three strategies. Experimental results are provided in Tables 2 and 3, where *S^O^* is calculated by Strategy OS-1 and Strategy OS-2, respectively.

It can be found from Tables 2 and 3 that the k-NN test performance is significantly improved by introducing the S^I^ feature, no matter how many neighbors are used.

#### 4.3.2. Effect of the Final Match Score

**Figure 10.**
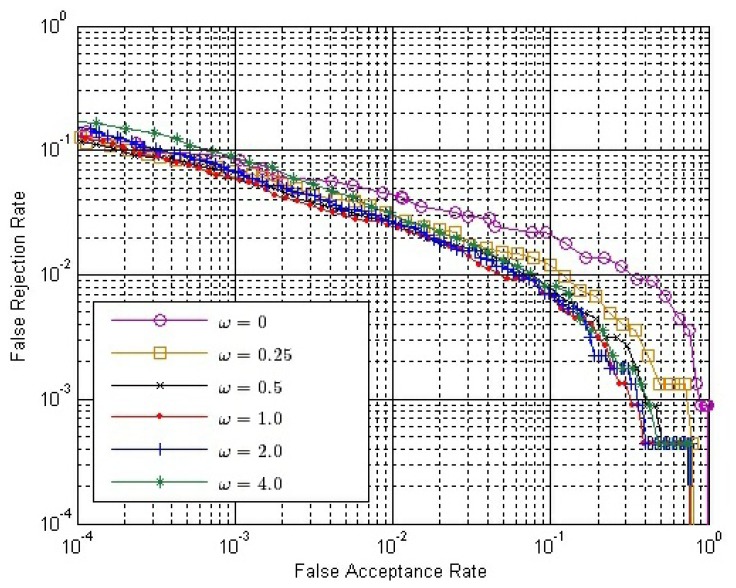
ROC of the final match score with different *ω* values, where both *S^E^* and *S^C^* are calculated by Strategy IS-2 and *S^O^* is calculated by Strategy OS-1.

**Figure 11.**
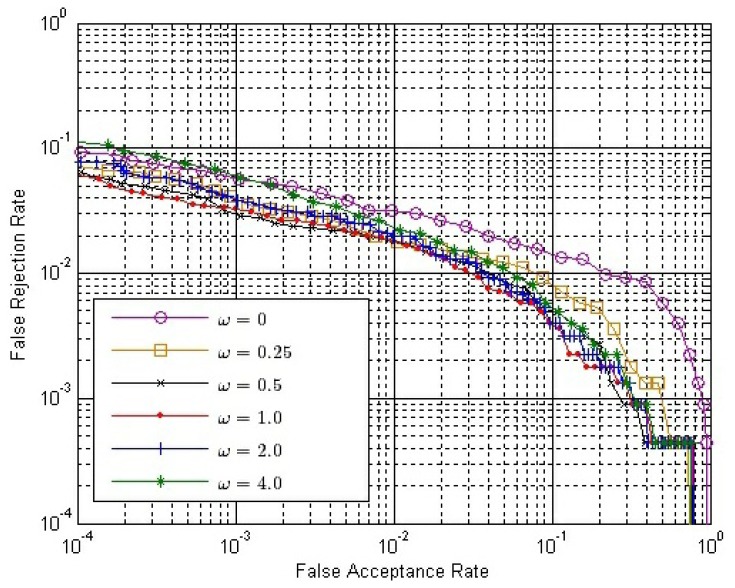
ROC of the final match score with different *ω* values, where both *S^E^* and *S^C^* are calculated by Strategy IS-2 and *S^O^* is calculated by Strategy OS-2.

The final match score between two matching fingerprint videos is calculated by Equation (14). Therefore, we have to determine the value of *ω*. From Section 3.4, we can conclude that *ω* should be no less than zero or it will have an adverse effect to verification. If *ω* = 0, only *S^O^* is used. Here, we choose six possible values of *ω*, changing from zero to four. Figure 10 shows the ROC of the final match score, *S*, with different *ω* values, where both *S^E^* and *S^C^* are calculated by Strategy IS-2 and *S^O^* is calculated by Strategy OS-1, while Figure 11 shows the ROC of *S* using the same strategies, except that *S^O^* is calculated by Strategy OS-2.

In both Figures 10 and 11, the performance is improved gradually, with the *ω* value increased from zero to one; while the performance is degraded gradually, with the *ω* value increased from one to four. Therefore, we can conclude that the performance of our method is affected by the value of *ω*, and the approximately best performance will be achieved when *ω* is around one.

**Figure 12.**
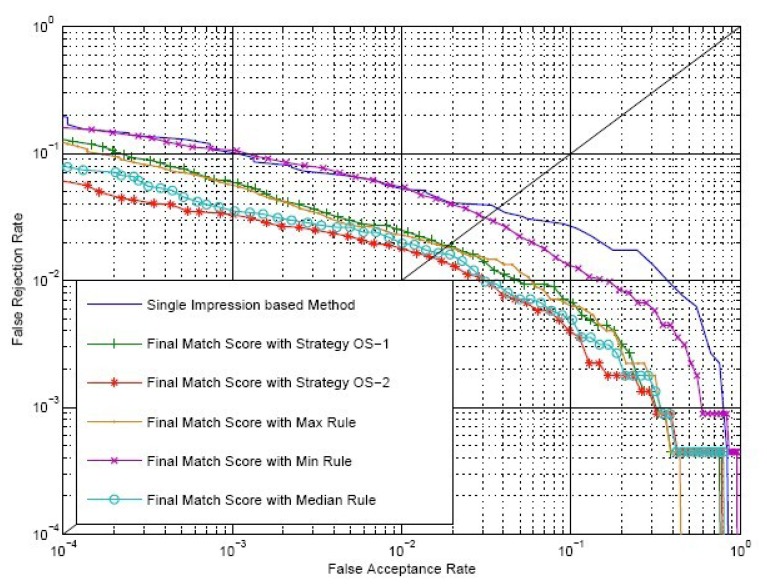
ROC of the final match score of our method with *ω* = 1.0, maximum rule, minimum rule, media rule and the conventional single impression-based method for comparison.

We select *ω* = 1.0 and make comparisons between the conventional single impression-based method and our method. *S^O^* is calculated by Strategy OS-1, Strategy OS-2, maximum rule, minimum rule and median rule. *S^I^* is calculated by Equation (9), where both *S^E^* and *S^C^* are calculated by Strategy IS-2. ROC of the conventional method and our method are shown in Figure 12. EER of the conventional method, our method using Strategy OS-1, Strategy OS-2, maximum rule, minimum rule and media rule are 3.65%, 1.92%, 1.50%, 1.83%, 3.07% and 1.65%, respectively. Therefore, there is a relative reduction of 60 percent in the EER when the fingerprint video is introduced for verification. As there exists strong correlation between impressions inside a fingerprint video, using only outside similarity for verification leads to a minor improvement. However, if we take advantage of the correlation between impressions to define inside similarity and use both outside similarity and inside similarity for verification, a significant improvement will be achieved.

#### 4.3.3. Runtime

**Figure 13.**
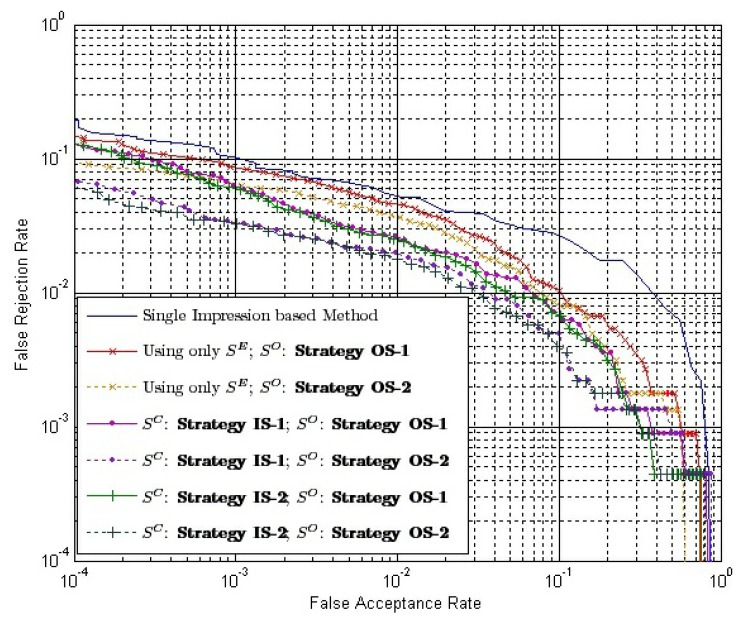
ROC of the final match score using different combinations of strategies with *ω* = 1.0 and the conventional method using single impressions for comparison.

Suppose the time complexity of calculating a one-on-one matching between two impressions is *O*(1); then, the time complexity of calculating *S^C^* using Strategy IS-1 and Strategy IS-2 is *O*(*k*) and *O*(*k*^2^), respectively, while the complexity of calculating *S^O^* using Strategy OS-1 and Strategy OS-2 is *O*(1) and *O*(*k*), respectively, where *k* is the number of impressions in each of the aligned matching videos. Note that the calculation of *S^E^* is offline. We can use only *S^E^* to calculate inside similarity, *i.e.*, using Equation (8). Therefore, the time complexity of calculating inside similarity will be reduced to *O*(1). Table 4 provides the time complexity of different combinations of strategies to calculate the final match score. Figure 13 shows the ROC of the final match score using these combinations with *ω* = 1.0.

From the ROC, we can conclude that:

(1) Our method outperforms the conventional method, even if the time complexities of both methods are equal to *O*(1), *i.e.*, the inside similarity using only *S^E^* and the outside similarity using Strategy OS-1.
(2) When inside similarity is calculated by the same strategy, the final match score using Strategy OS-1 to calculate outside similarity outperforms that using Strategy OS-2.
(3) When outside similarity is calculated by the same strategy, the final match score using both *S^E^* and *S^C^* outperforms that using only *S^E^*.
(4) When outside similarity is calculated by the same strategy, using Strategy IS-1 or Strategy IS-2 to calculate *S^C^* leads to almost the same performance.

Another factor affecting the runtime is the value of *k*. Suppose the average number of impressions in every matching video is *k̄*; then, the value of *k̄* is six after aligning. We can change the number of impressions in every aligned video to get a larger or smaller value of *k̄* from six.

The *k̄* values are expected to be four, eight and 8.8, which is the largest value that *k̄* can achieve. Therefore, we try to abandon two impressions (not including the impression with the largest fingerprint area; at the beginning of every aligned video to let the *k̄* value be four. We also try to reintroduce two conjoint impressions, which are abandoned during the aligning procedure to let the *k̄* value be eight. Finally, we use all the remaining impressions in the preprocessed video. After these three aspects of processing, the *k̄* value becomes 4.2, 7.1 and 8.8, respectively.

**Figure 14.**
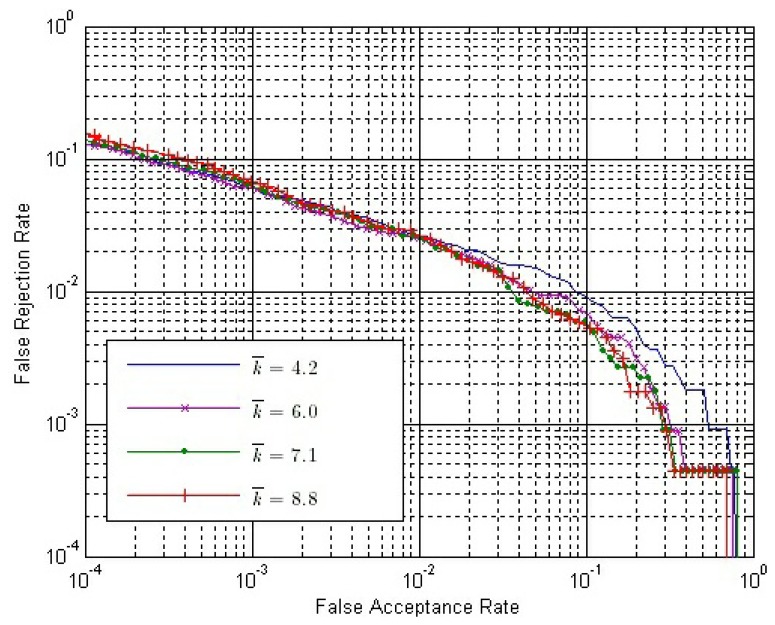
ROC of the final match score with different *k̄* values, where both *S^E^* and *S^C^* are calculated by Strategy IS-2 and *S^O^* is calculated by Strategy OS-1.

**Figure 15.**
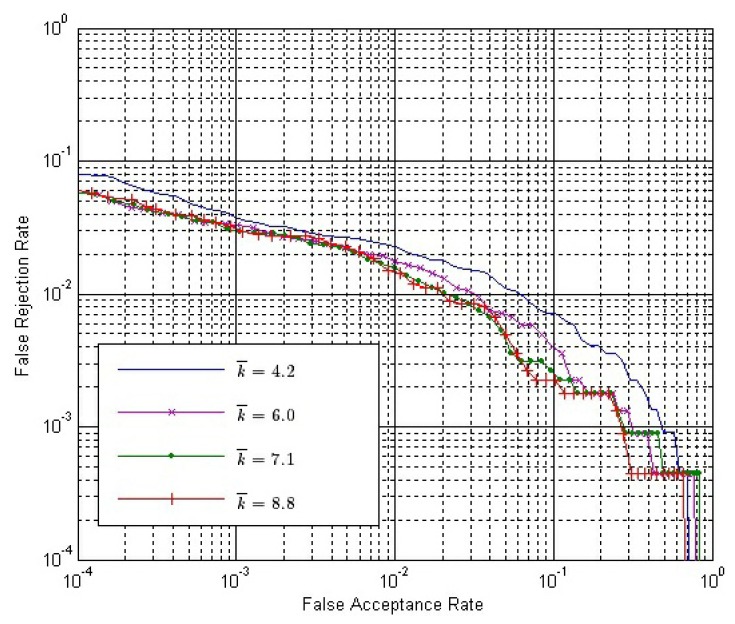
ROC of the final match score with different *k̄* values, where both *S^E^* and *S^C^* are calculated by Strategy IS-2 and *S^O^* is calculated by Strategy OS-2.

Let both *S^E^* and *S^C^* be calculated by Strategy IS-2 and *ω* = 1.0. The ROC of the final match score with different *k̄* values are shown in Figures 14 and 15, where *S^O^* is calculated by Strategy OS-1 and Strategy OS-2, respectively.

From Figures 14 and 15, we can conclude that:

(1) With the same *k̄* value, the performance is better if Strategy OS-2 rather than Strategy OS-1 is used to calculate *S^O^*.
(2) In both figures, the performance is gradually improved, with the *k̄* value getting larger. However, the improvement is gradually weaker, and the performance is almost the same when *k̄* is 7.1 and 8.8.
(3) The improvement by using a larger *k̄* value is more apparent if the Strategy OS-2 is used to calculate *S^O^*.

#### 4.3.4. Comparisons with the Fusion of Multiple Impressions

The calculation of outside similarity uses the same idea with the fusion of multiple impressions from the same finger. However, the proposed video-based method is quite different with the multiple impressions fusion method, because the most important part of our method is taking advantage of the dynamic information, *i.e.*, strong correlation between impressions inside a fingerprint video. Additionally, experimental results show that the largest part of the accuracy improvement is due to the introduction of inside similarity. In this section, new experiments are designed and carried out to make comparisons between the proposed video-based method and the multiple impressions fusion method.

Suppose the individuals in the SDU-FV database are represented by *I_j_* (*j* = 1, 2, …, 50), and the fingerprint videos from individual *I_j_* are represented by *V_j_*,*_i_* (*i* = 1, 2, …, 10). We will select three templates for fusion in the multiple impressions fusion method. We separate the ten fingerprint videos of each individual, *I_j_*, to three groups. Group 1 consists of *V_j_*_,1_, *V_j_*_,2_ and *V_j_*_,3_; group 2 consists of *V_j_*_,4_, *V_j_*_,5_ and *V_j_*_,6_; the remaining videos belong to group 3.

**Figure 16.**
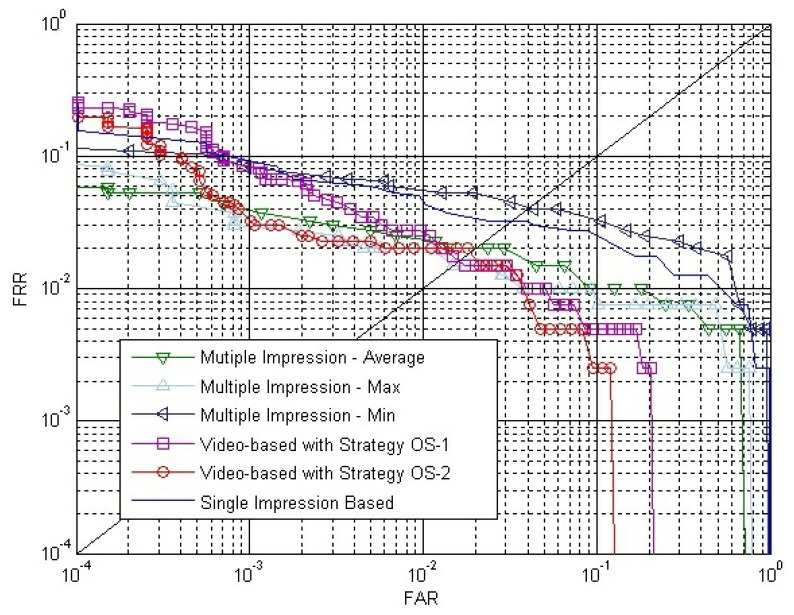
ROC of the single impression-based method, the multiple impressions fusion method and the proposed video-based method.

**Figure 17.**
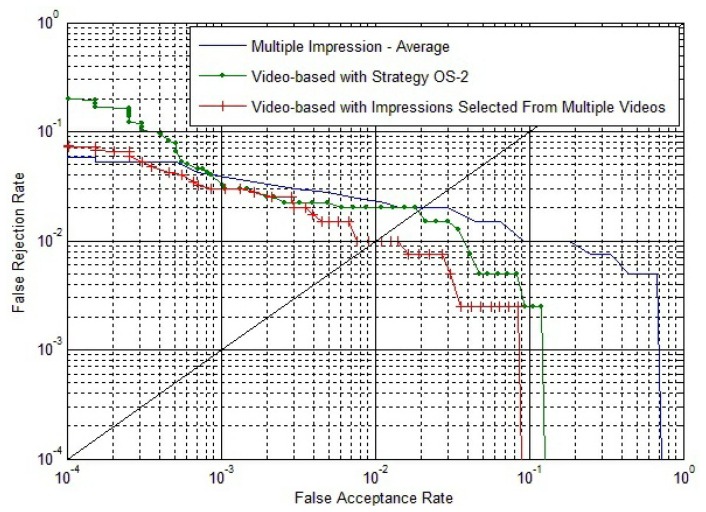
ROC of the multiple impressions fusion method, the proposed video-based method and the video-based method with impressions selected from multiple videos.

(1) Experiments of the multiple impressions fusion method:
  From Figure 9, we conclude that using the impression with the largest fingerprint area in a fingerprint video can access better performance. For individual *I_j_*, the impressions with the largest fingerprint area in *V_j_*_,1_, *V_j_*_,2_ and *V_j_*_,3_ respectively, are selected as three templates. The impression with the largest fingerprint area in *V_k_*,*_l_* (*k* = 1, 2, …, 50; 7 ≤ *l* ≤ 10) is selected as the claimed impression. Firstly, the three templates match against the claimed impression, and three match scores are calculated. Secondly, we take the average, maximum and minimum of the three match scores, respectively, as the score-level fusion result. Therefore, there are 4 × 50 = 200 genuine matches and 4 × 49 × 50 = 9800 impostor matches. Similarly, the impressions with the largest fingerprint area in *V_j_*_,4_, *V_j_*_,5_ and *V_j_*_,6_, respectively, are selected as three templates. The following steps are the same as described above. Therefore, the total number of matches are 20,000, with 2 × 200 = 400 genuine matches and 2 × 9800 = 19,600 impostor matches.
(2) Experiments of the proposed video-based method:
  Individual *I_j_*, *V_j_*_,1_ and *V_j_*_,4_ are selected as enrolled videos, respectively, and *V_k_*,*_l_* (*k* = 1, 2, …, 50; 7 ≤ l ≤ 10) is selected as the claimed video. Therefore, the number of genuine matches and impostor matches are the same as that in the experiments of the multiple impressions fusion method. Both *S^E^* and *S^C^* are calculated by Strategy IS-2.
  Besides, we also carry out an experiment of single impression-based matching, which uses the impression with the largest fingerprint area in a fingerprint video. The results of all these experiments are shown in Figure 16.
  From Figure 16, we can conclude that the video-based method can lead to better accuracy than the multiple impressions fusion method, especially since the proposed method gets much lower FAR when FRR is quite low.
(3) Experiments of the video-based method with impressions selected from multiple videos:
  The impression with the largest foreground size from *V_j_*_,1_, *V_j_*_,2_ and *V_j_*_,3_, respectively, are selected to compose a template fingerprint video, *FV_j_*_,1_. *FV_j_*_,1_ will match against each fingerprint video in group 3. Similarly, the impression with the largest foreground size from *V_j_*_,4_, *V_j_*_,5_ and *V_j_*_,6_ is selected to compose a template fingerprint video, *FV_j_*_,2_, to match against each fingerprint video in group 3. Therefore, there will be eight genuine matches for each individual. And there will be 400 genuine matches for all the 50 individuals. For the impostor matches, *FV_j_*_,1_ and *FV_j_*_,2_ will match against other individuals' fingerprint video in group 3. Therefore, there will be 50 × 49 × 8 = 19,600 impostor matches.
  Experiments of the video-based method with impressions selected from multiple fingerprint videos are carried out. For comparison, experimental results of multiple impression matches and fingerprint video matches that have been described above are also shown in Figure 17.

The EER of the multiple impression method, the video-based method with Strategy OS-2 and the video-based method with impressions selected from multiple videos are, respectively, 2.0%, 1.9% and 1.0%. We can conclude that the proposed video-based method can access a much better result than the multiple impression fusion method.

The video-based method with impressions selected from multiple videos leads to better accuracy than the video-based method with Strategy OS-2. This is because impressions with the largest foreground size in each video are selected to compose a template video. The fingerprint image quality is much better than the impressions selected from the same video. Additionally, the similarity of fingerprint videos acquired from the same individual are quite high, as there is no significant plastic distortions in the acquiring process.

**Figure 18.**
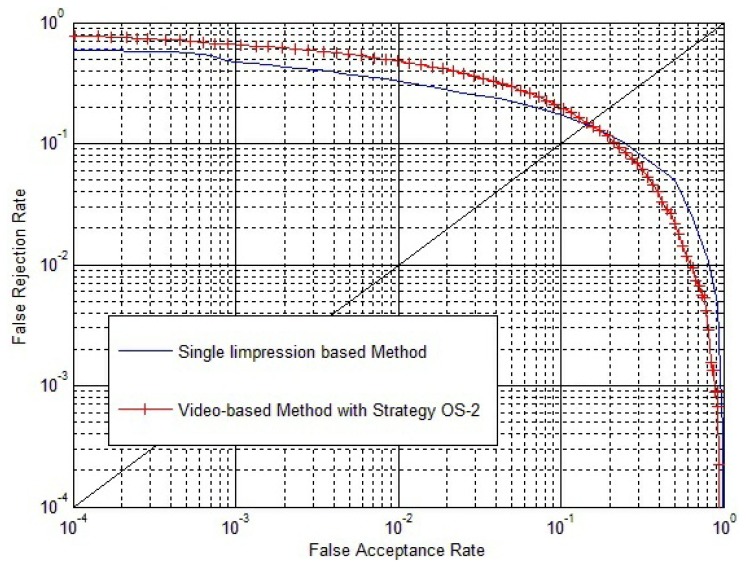
ROC of the single impression-based method and the video-based method with Strategy OS-2.

Significant plastic distortions in the fingerprint video will lead to quite low inside similarity of the fingerprint video. To improve that, since if all the fingerprint videos have quite a low inside similarity, the video-based method will no longer be effective, we carried out experiments on the fingerprint video database, NIST 24. Experimental results are shown in Figure 18.

From Figure 18, we can conclude that if there are significant plastic distortions in fingerprint videos that lead to quite low inside similarity for all the fingerprint videos, the video-based method will no longer be effective. This confirms our argument in Section 3.4: “We have to notice that the foundation of this method is that the match score between two impressions in the same fingerprint video is quite high, due to their strong correlation, and *S^I^* is an approximate representation of the maximum value of $S_{g}^{O}$”.

## 5. Conclusions and Future Work

We proposed to utilize videos for fingerprint verification. After preprocessing and aligning processes, “inside similarity” and “outside similarity” were defined to take advantage of dynamic and static information contained in fingerprint videos. Then, the match score between two matching fingerprint videos was calculated by combining the two kinds of similarity. In fact, the proposed video-based method is a wrapped method that is based on one-on-one matching. Experimental results show that the video-based method leads to a significant accuracy improvement in comparison to the conventional single impression-based method. More importantly, our method outperforms the conventional method, even if the time complexities of both methods are equal. Besides, experimental results also demonstrate that the proposed video-based method outperforms the multiple impressions fusion method. Therefore, fingerprint video is more informative and has higher accuracy.

Future work includes selecting an optimized equation to calculate the match score of two matching fingerprint videos. Additionally, currently, we are exploring more useful information from fingerprint video for verification and acquiring a larger database for testing. We are also investigating the potential of alleviating security issues by using fingerprint videos.

## Figures and Tables

**Table 1.** An example that shows the benefits of using the *relative match score* for verification. We can get the correct verification result if *SO* − *S^I^* is used, while we cannot if *S^O^* is used. (*S^O^* and *S^I^* are all from zero to 100).

	***S****^O^*	***S****^I^*	***S****^O^***−*S****^I^*
A genuine match	25	80	−55
An impostor match	30	90	−60

**Table 2.** The *k*-nearest neighbor (*k*-NN) error rates (%) of using the *S^O^* feature only and using two-dimensional features, *i.e.*, (*S^I^*, *S^O^*), respectively. *S^E^* is calculated by Strategy IS-2, and *S^O^* is calculated by Strategy OS-1.

***k*values**	**1**	**5**	**9**	**13**	**17**
**strategies**					
*S^O^*	69.51	43.65	17.66	7.484	2.462
(*S^O^*, *S^I^*), using only *S^E^*	2.353	0.1804	0.1740	0.1772	0.1780
(*S^O^*, *S^I^*), *S^C^*: Strategy IS-1	0.4522	0.1989	0.1956	0.1900	0.1892
(*S^O^*, *S^I^*), *S^C^*: Strategy IS-2	0.4346	0.1924	0.1908	0.1868	0.1804

**Table 3.** The k-NN error rates (%) of using the *S^O^* feature only and using two-dimensional features, i.e., (*S^I^*, *S^O^*), respectively. *S^E^* is calculated by Strategy IS-2 and *S^O^* is calculated by Strategy OS-2.

***k*Values**	**1**	**5**	**9**	**13**	**17**
**strategies**					
*S^O^*	0.9293	0.1748	0.1692	0.1684	0.1636
(*S^O^*, *S^I^*), using only *S^E^*	0.4145	0.1187	0.1195	0.1179	0.1203
(*S^O^*, *S^I^*), *S^C^*: Strategy IS-1	0.3640	0.1107	0.1074	0.1082	0.1050
(*S^O^*, *S^I^*), *S^C^*: Strategy IS-2	0.3632	0.1107	0.1066	0.1050	0.1058

**Table 4.** Different combinations of strategies to calculate the final match score and their time complexities. *S^E^* is calculated by Strategy IS-2, and the computation is offline.

**Inside similarity**	**Using only*S****^E^***(offline)**	***S****^C^***:Strategy IS-1 (*O*(*k*))**	***S****^C^***:Strategy IS-2 (*O*(*k*^2^))**
**Outside similarity**			
Strategy OS-1 (*O*(1))	*O*(1)	*O*(1) + *O*(*k*)	*O*(1) + *O*(*k*^2^)
Strategy OS-2 (*O*(2*k*))	O(2*k*)	O(2*k*) + O(*k*)	O(2*k*) + O(*k*^2^)
